# Survey and detection for citrus tristeza virus in Florida groves with an unconventional tool: The Asian citrus psyllid

**DOI:** 10.3389/fpls.2022.1050650

**Published:** 2022-12-07

**Authors:** Kellee Britt-Ugartemendia, Donielle Turner, Peggy Sieburth, Ozgur Batuman, Amit Levy

**Affiliations:** ^1^ Department of Plant Pathology, Southwest Florida Research and Education Center, University of Florida, Immokalee, FL, United States; ^2^ Department of Plant Pathology, Citrus Research and Education Center, University of Florida, Lake Alfred, FL, United States

**Keywords:** Diaphorina citri, tristeza disease, CTV strains, bioindicator, Huanglongbing, ACP

## Abstract

The citrus industry of Florida faces insurmountable challenges against the destructive diseases citrus tristeza and Huanglongbing (HLB, or citrus greening). Though the tristeza causal agent, citrus tristeza virus (CTV), has been in Florida decades longer than HLB, growers have concentrated most of their efforts on combating the more detrimental HLB. The Asian citrus psyllid (*Diaphorina citri*; ACP) is the insect vector of the bacterial pathogen *Candidatus* Liberibacter asiaticus and transmits the incurable HLB to all commercial citrus. During our searches for biological and viral controls against the ACP, we consistently detected sequences of CTV in Florida field populations of ACP. This unexpected finding led us to investigate whether ACPs collected from young shoots could be used as a tool to survey CTV in Florida citrus groves. We first surveyed for the most common CTV strains in Florida (T30, T36, and VT/T68) in citrus trees on mostly sour orange (*Citrus aurantium*) rootstock, the rootstock susceptible to CTV decline. Out of 968 trees sampled across five years (2018-2022), approximately 8.2% were positive for CTV, with more than half of the CTV-positive trees infected with strain T30. Simultaneously, we looked at CTV strains in ACPs during this time and found that approximately 88% of pooled adult and nymph ACPs also had CTV, with over half the positive samples having the T36 strain. As a result of the much higher CTV incidences in the ACPs, we conducted a second investigation into whether we could more easily detect the same CTV strains in ACP nymphs as in CTV-infected citrus tissue. After individually sampling 43 trees and pooling the nymphs from each tree, we detected CTV at about the same incidence in the citrus tissue and the nymphs, but with much less ACP tissue, time, and resources required for detection compared to citrus tissue. Results from this study illustrate the sustained threat of CTV to Florida citrus and demonstrate the ACP as a potential bioindicator for CTV.

## Introduction

The largest known closterovirus, citrus tristeza virus (CTV; Family *Closteroviridae*, Genus *Closterovirus*), is currently the most economically damaging citrus virus in the world and remains a particular threat to Florida citrus (Hilf and Garnsey, 2002; Folimonova and Sun, 2022). CTV is phloem-limited, can infect all commercial citrus, and is vectored by aphids (Hemiptera: *Aphididae*) (Norman and Grant, 1956; Simanton and Knorr, 1969; Bar-Joseph et al., 1979; Roistacher and Bar-Joseph, 1987; Bar-Joseph et al., 1989). CTV can be devastating in sweet orange (*Citrus sinensis*) trees grafted onto sour orange (*Citrus aurantium*), a historically popular rootstock for its exceptional traits (Bowman et al., 2021). However, following the initial detection of CTV in Florida in 1952 (Grant, 1952), the distribution and diversity of the virus continue to pose significant challenges to citrus in the state (Hilf and Garnsey, 2002). The susceptibility of sour orange rootstock to CTV-decline during the latter half of the twentieth century drastically reduced its use in Florida (Bowman et al., 2021).

Three major CTV strains currently exist throughout Florida citrus-producing regions: T30, known as the “mild strain”, T36, known as the “decline” strain, and finally VT, a strain that can also cause decline and stem pitting (Dawson et al., 2013; Harper et al., 2015b; Harper and Cowell, 2016). Infections do not always manifest symptoms as expected depending on tree rootstock/scion combination, and mixed CTV populations of these strains can interfere with visual diagnoses (Cohen and Knorr, 1953; Bar-Joseph et al., 1989; Harper and Cowell, 2016). The CTV T30 “mild strain” mostly induces mild symptoms in diseased citrus, such as slight stunting of the tree, and can even be asymptomatic, which allows for the continual productivity of the infected citrus tree for several years (Lee and Keremane, 2013). On the other hand, the T36 “quick decline” strain of CTV can rapidly weaken and kill an infected citrus tree on sour orange rootstock in just a few weeks but may take up to two years, depending on isolate virulence (Lee and Keremane, 2013). VT and T68 strains can be as damaging to infected citrus as T36 strains but are not as widely distributed throughout Florida citrus groves as T36 and have not historically imposed as much of a threat (Harper and Cowell, 2016). Mild stem pitting phenotypes of the genetically similar VT and T68 strains have been found scattered throughout the state, though the Florida citrus industry continues to evade severe isolate epidemics (Roy and Brlansky, 2010; Harper, 2013).

Florida citrus growers are also struggling against the endemic and incurable disease Huanglongbing (HLB, or citrus greening) since its detection in 2005 (Halbert, 2005; Bové, 2006). Growers largely depend on chemical controls for the Asian citrus psyllid (*Diaphorina citri*; ACP), a hemipteran, phloem-feeding insect and the primary dispersal agent of the HLB bacterium, *Candidatus* Liberibacter asiaticus (*C*Las) (Hall et al., 2013). Cases of insecticide resistance of the ACP (Tiwari et al., 2011; Kanga et al., 2016) emphasize a need for more diverse management options in Florida. While studying insect-specific viruses (ISVs) in Florida ACP populations to address this, we unearthed a consistent occurrence of CTV sequences in the ACP (Britt et al., 2020; Britt et al., 2022). The incomplete genomic coverage of CTV detected in the ACP suggested that the citrus virus was likely consumed as a part of the diet when feeding on CTV-infected citrus (Britt et al., 2022). Phloem contents (and any microbes present) may accumulate in the gut of the insect. The fact that ACP nymphs feed entirely on flush tissue after hatching from eggs, where CTV can be at its highest titer in the infected tree (Garnsey et al., 1979; Halbert and Manjunath, 2004), offers a unique and almost targeted way to sample the phloem contents of a tree for CTV. Thus, most, if not all, of the contents of the ACP nymph gut is citrus phloem.

In this study, we conducted two separate investigations: (1) survey the current status and diversity of CTV populations throughout Florida citrus groves on predominantly sour orange rootstock using either citrus tissue or ACPs, and (2) compare CTV detection directly in citrus tissue versus indirectly in feeding ACP nymphs. We hypothesize that the constantly feeding ACP nymphs would offer increased access to phloem contents—and possibly CTV—than the harder-to-reach phloem of citrus tissue during extraction and CTV testing.

## Materials and methods

### Citrus tristeza virus survey

#### Plant and insect tissue

**Figure 1:**
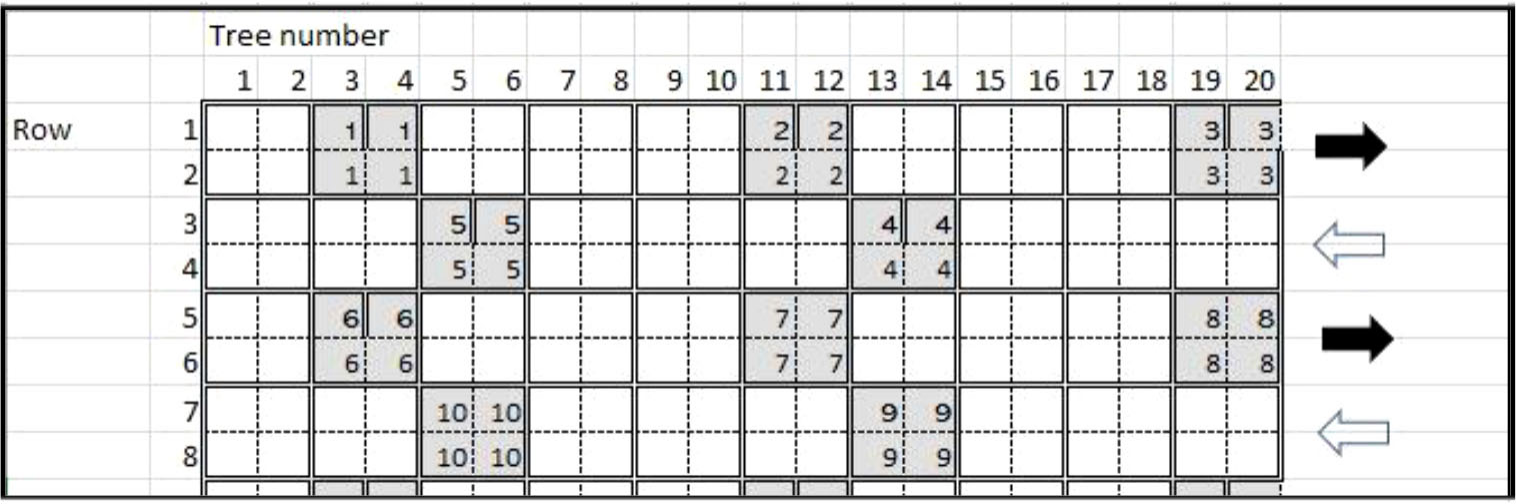
Grid of tree hierarchal sampling scheme for citrus tristeza virus throughout Florida citrus groves during 5 years (2018-2022) of the initial survey. The numbers within the gray boxes indicate samples of tree sets. The black and white arrows indicate the direction of travel through the grove and the location of the trees.

**Figure 2:**
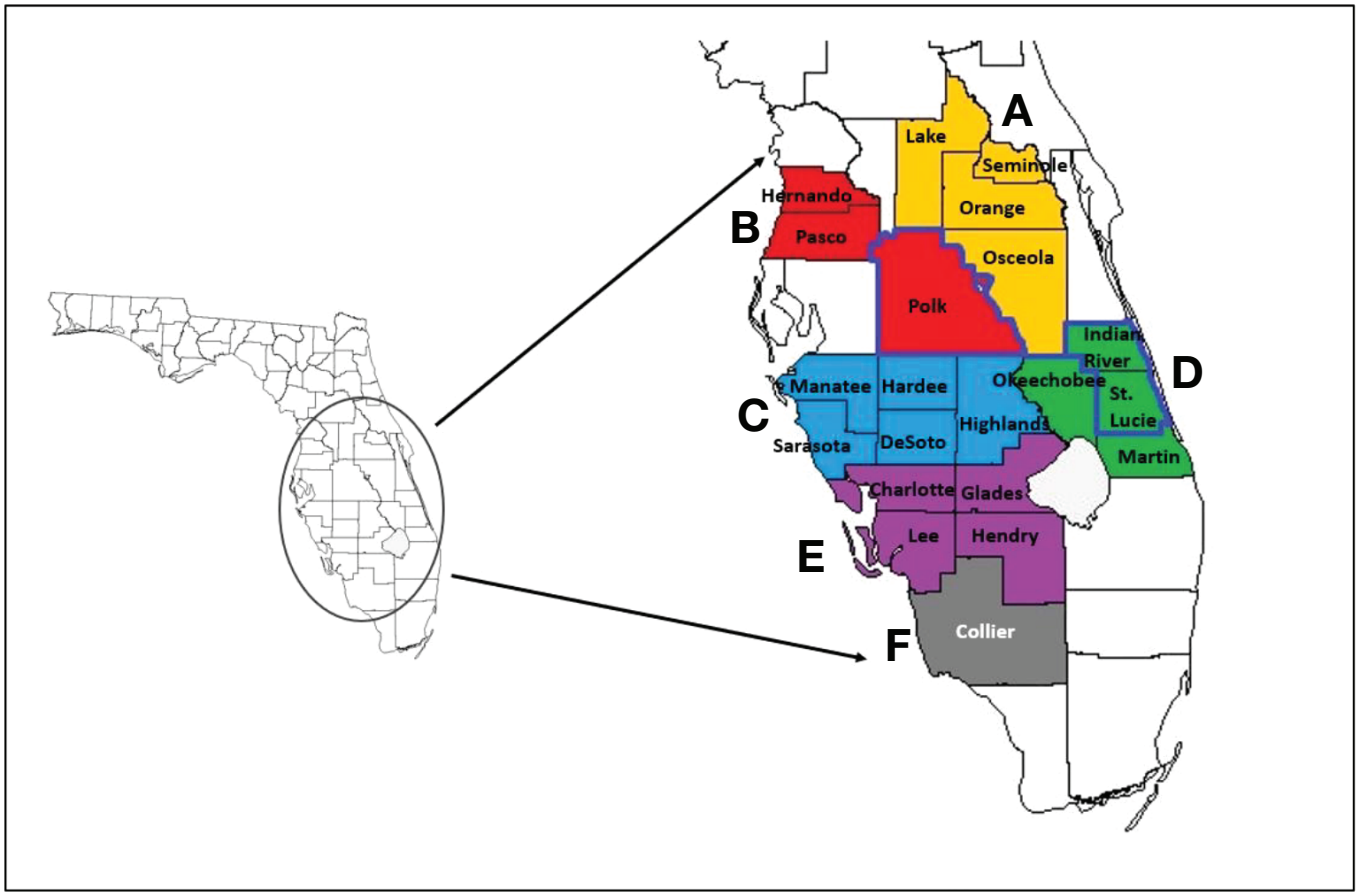
Map of Florida and counties included for citrus and Asian citrus psyllid (ACP) sampling. The enlarged counties of the same color are in the same region (regions A-F) and represent major citrus-producing counties of Florida pooled for regional ACP samples. Counties outlined in darker blue (Polk County, Indian River County, and St. Lucie County) represent the counties visited for only citrus samples. Image adapted from Britt et al. (2020).

*Plant*. For surveying current CTV diversity in Florida citrus groves, initial survey samples were collected from a four-tree block in each grove based on the hierarchal sampling scheme described by Gottwald and Hughes (2000) (**Figure 1**). Samples were comprised of fully expanded flush leaves that had not hardened off from the tree. Groves were located in three major Florida citrus-producing counties: Indian River, Polk, and St. Lucie (**Figure 2**). Flush leaves were taken from 5-8 locations on any individual tree, and four trees were pooled into a single sample.

*Psyllid*. Adult and nymph Asian citrus psyllids (ACPs) were collected and processed similar to previously described (Britt et al., 2020). Briefly, young flushes colonized with nymphs and/or adults were scouted in citrus groves throughout the major citrus-producing regions and counties in Florida between 2018 to 2022, totaling 24 pooled samples. These regions, A-F, and their Florida counties have been previously organized by Britt et al. (2020). Region A was composed of Lake, Seminole, Orange, Osceola counties; Region B, Pasco, Hernando, and Polk; Region C, Manatee, Hardee, Highlands, DeSoto, and Sarasota; Region D, Indian River, Okeechobee, St. Lucie, and Martin; Region E, Charlotte, Glades, Lee, and Hendry; Region F, Collier County (**Figure 2**). Live ACP nymphs and adults were collected and the flush leaves they were feeding on were immediately submerged in 100% ethanol to kill and preserve the insects. ACPs were separated out from the flush leaves, counted with the aid of a stereo microscope (Leica S8 APO, Leica Microsystems, USA), and stored separately in smaller vials containing 100% ethanol at -20°C until needed.

#### Total nucleic acid extraction and real-time PCR

*Plant*. For the survey, 250 mg of midrib and/or petiole tissue from each sample were chopped into small pieces with a razorblade sterilized with 20% bleach, and placed into a 2 ml microcentrifuge tube along with two 4.5 mm steel beads (Daisy BBs, Rogers, AR, USA). The tubes are then placed in liquid nitrogen for 5 minutes. Subsequently, the tissue was ground into a fine powder with a Retsch TissueLyser II (Qiagen, Hilden, Germany) at 30 beats/sec for 30 seconds. Total nucleic acid (TNA) was then extracted from the sample using TRIzol™ Reagent (Thermo Fisher Scientific, Waltham, MA, USA) according to the manufacturer’s instructions. The resulting TNA was then checked for purity and concentration with a BioDrop spectrophotometer (BioDrop, Cambridge, UK).

*Psyllid*. Preparation for RNA and DNA (total nucleic acid, TNA) extraction from nymph and adult ACPs was done similar to previously described (Britt et al., 2020). Briefly, approximately 5-60 adult and/or nymph ACPs (depending on availability) were pooled and placed into a sterile 1.5 ml microcentrifuge tube with three 2.3 mm chrome steel beads (BioSpec Products Inc, Bartlesville, OK, USA). The tubes were sealed with Breathe-Easy tube membranes (Genesee Scientific, CA, USA), placed into a Labconco FreeZone Freeze-Dry System (Kansas City, MO, USA) overnight and ground in a Mini-Beadbeater™ (BioSpec Products Inc, Bartlesville, OK, USA) to a fine powder. All pulverized insect samples were subjected to TNA extraction using TRIzol™ Reagent (Thermo Fisher Scientific, Waltham, MA, USA) according to the manufacturer’s instructions or using the potassium acetate-SDS (KAc-SDS) extraction method. The nucleic acid extracted was measured for purity and quantity with a Synergy HTX plate reader (BioTek Instruments, Winooski, VT, USA).

*Real-time PCR*. One μl of extracted TNA from each sample (100-125 ng/μl) (technical duplicates) was used in each real-time PCR reaction. All PCRs were completed in duplexed 12-µl reactions and were modified from Harper et al. (2014; 2015a). For citrus tissue, reactions consisted of 11-µl mix of TaqMan™ RNA-to-CT™ 1-Step Kit (Applied Biosystems, Life Technologies, Carlsbad, CA, USA) with approximately 1 μM of each CTV primer and 150 nM of CTV probe, and 1 μM of each citrus internal control primer and 150 nM of probe (**Table 1**). For ACP tissue, the same amount of PCR mix was used along with approximately 1 μM of each CTV primer and 150 nM of probe, and 1 μM of each ACP internal control primer and 150 nM of probe (**Table 1**). CTV primers target an approximately 90-101 base-pair region of the CTV coat protein (*p25*) gene, depending on the primer set (Saponari et al., 2008; Harper et al., 2014; Harper et al., 2015a). Real-time PCRs occurred in MicroAmp^®^ EnduraPlate™ Optical 96-Well Fast Clear Reaction Plates (Life Technologies, Thermo-Fischer Scientific, Carlsbad, CA, USA) and were completed in a 7500 Fast Real-Time PCR System (Applied Biosystems, Foster City, CA, USA). Each PCR run contained the following controls: one well with water as a no template control (NTC); one well positive for either the ACP or citrus target but negative for CTV; and one well positive for either the ACP or citrus target and CTV target. Negative nymph and adult ACP controls for CTV were taken from a laboratory colony reared on orange jasmine (*Murraya paniculata*). Negative citrus controls for CTV were taken from greenhouse-grown citrus plants maintained at the Southwest Florida Research and Education Center in Immokalee, FL. Citrus CTV-positive control was TNA extracted from a mandarin orange (*Citrus reticulata*) plant infected with CTV isolate FS703 (T30, T36, and VT). It was grown in a temperature-controlled greenhouse at the Citrus Research and Education Center (CREC) in Lake Alfred, FL, and originally gifted to us by the Florida Division of Plant Industry.

**Table 1:** List of primers and probes used for citrus tristeza virus (CTV) generic and strain-specific detection in citrus trees and Asian citrus psyllids (*Diaphorina citri*; ACP).

Primer or Probe	Nucleotide Sequence (5’ – 3’), Fluorophore and Quencher if labeled	Purpose	Reference
CTV generic (*p*25) (all strains) Forward	AGCRGTTAAGAGTTCATCATTRC	CTV detection	(Saponari et al., 2008)
CTV generic (*p*25) (all strains) Reverse	TCRGTCCAAAGTTTGTCAGA		
CTV generic (*p*25) (all strains) probe	6-FAM-CRCCACGGGYATAACGTACACTCGG-BHQ1		
Common Reverse for CTV strains	GCAAACATCTCGACTCAACTACC	CTV Strain-specific detection	(Harper et al., 2014; Harper et al., 2015a)
CTV T36 Forward	ACCTCGGACAAGCGGGTGAATT		
CTV T30, VT/T68 Forward	CGATGGTCAAGCGGACGACTT		
CTV T36 Probe	6-FAM-AGCAACCGGCTGATCGATTGATT-BHQ1		
CTV VT/T68 Probe	6-FAM-AGCGACAGGCTGATGGTTTGTTCA-BHQ1		
CTV T30 Probe	6-FAM-TGAACAAACGATCAACCAGTCATC-BHQ1		
*Dc wingless* gene Forward	TGGTGTAGATGGTTGTGATCTGATGTG	Internal quality control for ACP nucleic acid	(Manjunath et al., 2008)
*Dc wingless* gene Reverse	ACCGTTCCACGACGGTGA		
*Dc wingless* gene probe	6-JOE-TGTGGGCGAGGCTACAGAAC-BHQ1		
*COX* gene Forward	GTATGCCACGTCGCATTCCAGA	Internal quality control for citrus nucleic acid	(Li et al., 2006)
*COX* gene Reverse	GCCAAAACTGCTAAGGGCATTC		
*COX* gene probe	6-JOE-ATCCAGATGCTTACGCTGG-BHQ2		

p, protein; Dc, *Diaphorina citri*; COX, plant cytochrome oxidase.

The PCR cycling conditions consisted of a holding stage for reverse transcription at 48°C for 15 minutes, followed by another holding stage 95°C for 10 minutes, and then 40 cycles of 95°C for 15 seconds and 60°C for 1 minute. All samples and controls were tested in duplicate. The cycle threshold (Ct) value was calculated, and it was determined that any Ct value ≤ 36 was considered positive for CTV, and any Ct value > 36 as negative for CTV (Bertolini et al., 2008; Saponari et al., 2008; Kokane et al., 2021). To validate the strain-specific PCRs and verify their specificity without cross-reactivity, TNA was extracted from leaf flush midrib/petiole of citrus plants with single infections of T30, T36, T68, and VT (kindly provided by Dr. Svetlana Folimonova), as well as the citrus plant with CTV isolate FS703 (T30, T36, and VT) maintained at CREC. The TNA was then subjected to all strain-specific PCRs as described. All citrus and ACP nymph and/or adult TNA samples were initially screened with generic coat protein (*p*25) CTV primers (**Table 1**), then subjected to CTV strain-specific PCRs if positive. If or when any initial citrus tree samples tested positive for CTV, each tree in the infected sample(s) was individually tested (**Figure 1**) along with several of the neighboring trees by collecting flush leaves and extracting TNA as before. We again tested each sample using real-time PCR with the CTV strain-specific primers (**Table 1**).

### Analysis of citrus tristeza virus in citrus versus Asian citrus psyllid nymphs

#### Plant and insect tissue

Citrus leaf samples and ACP nymphs were collected from citrus groves in southwest Florida, Collier County, in spring (March to May) of 2022. **Table 2** describes the trees included in this part of the investigation. Trees that were sampled were randomly scouted and collected throughout groves, and had a range of mainly HLB symptoms, from very bare of leaves to full and healthy-looking canopies. Trees were sampled and tested individually. For each random tree chosen, 4-5 young flush bunches, preferably free of ACPs, were arbitrarily sampled across the tree and collected into a single sample. Simultaneously, ACP nymphs were similarly collected and pooled from the same tree. Live ACP nymphs and the flush leaves they were feeding on were immediately submerged in 100% ethanol to kill and preserve the insects. Nymph ACPs were subsequently separated out from the flush leaves, counted with the aid of a stereo microscope (Leica S8 APO, Leica Microsystems, USA), and stored separately in smaller vials containing 100% ethanol at -20°C until needed.

**Table 2:** Description of citrus tree and Asian citrus psyllid (*Diaphorina citri*; ACP) samples included in citrus tristeza virus (CTV) comparative study from Collier County, Florida.

Scion/Rootstock	Tree Sample ID	ACP Nymph Sample ID
Sweet orange ‘Valencia’ (*Citrus sinensis*)/Sour orange (*Citrus aurantium*)	T 1 – T 11	A 1 – A 11
Sweet orange ‘Valencia’/Swingle citrumelo [Duncan grapefruit (*Citrus paradisi* ‘Macfadyen’) × Trifoliate orange (*Poncirus trifoliata*)]	T 12 – T 15	A 12 – A 15
Duncan grapefruit/Kuharske (Sweet orange × Trifoliate orange)	T 16 – T 21	A 16 – A 21
Sweet orange¹/Various hybrids²	T 22 – T 33	A 22 – A 33
Sweet orange ‘Valencia’/Sour orange	T 34 – T 43	A 34 – A 43
**Total number of trees monitored: 43**		

T, Tree; A, ACP; ¹Mixture of cultivars: Hamlin and Valencia; ²Wilking mandarin × Trifoliate orange, Cleopatra mandarin × Swingle trifoliate, Sun Chu Sha mandarin, Sunki mandarin × Swingle trifoliate.

#### Total nucleic acid extraction and real-time PCR

*Plant*. All flush leaves were carefully inspected for any ACP eggs, nymphs, or adults, gently washed with deionized water, and dried with Kimwipes before processing for total TNA extraction. 100 mg of leaf flush (stem, leaf petiole/midrib) was finely chopped using a sterile razorblade and placed into a sterile 1.5 ml microcentrifuge tube with three 2.3 mm chrome steel beads (BioSpec Products Inc, Bartlesville, OK, USA). The tubes were sealed with Breathe-Easy tube membranes (Genesee Scientific, CA, USA), placed into a Labconco FreeZone Freeze-Dry System (Kansas City, MO, USA) overnight and ground in a Mini-Beadbeater™ (BioSpec Products Inc, Bartlesville, OK, USA) to a fine powder. Each tree sampled had biological duplicates from all quadrants of the tree.

*Psyllid*. Preparation for TNA extraction from only nymph ACPs was done similar to previously described (Britt et al., 2020). Adults ACPs were not used for this part of the study because they feed on multiple trees, confounding specific tree results. From each tree, five 4^th^-5^th^ instar nymphs or ten 1^st^-3^rd^ instar nymphs (<1 mg), depending on availability, were pooled and placed into a sterile 1.5 ml microcentrifuge tube with three 2.3 mm chrome steel beads (BioSpec Products Inc, Bartlesville, OK, USA). The tubes were sealed with Breathe-Easy tube membranes (Genesee Scientific, CA, USA), placed into a Labconco FreeZone Freeze-Dry System (Kansas City, MO, USA) overnight and ground in a Mini-Beadbeater™ (BioSpec Products Inc, Bartlesville, OK, USA) to a fine powder. Each tree sampled had biological duplicates of pooled nymphs tested from all quadrants of the tree similar to leaf flush. All pulverized plant and insect samples were subjected to TNA extraction using TRIzol™ Reagent (Thermo Fisher Scientific, Waltham, MA, USA) according to the manufacturer’s instructions.

Following extraction, plant TNA samples were diluted (1:50) to reduce inhibitory substances and verified of sufficient quantity and purity for real-time PCR (40-300 ng/μl) with a Synergy HTX plate reader (BioTek Instruments, Winooski, VT, USA). Psyllid TNA samples were also quantified (40-400 ng/μl) and verified of sufficient quality for real-time PCR.

*Real-time PCR*. PCRs were conducted the same as previously described for the initial CTV survey.

## Results

### Citrus tristeza virus survey

During a 5-year (2018-2022) survey, we sampled a total of 968 trees across three different major citrus-producing counties in Florida: Indian River, Polk, and St. Lucie (**Figure 2**; **Table 3**). Eight hundred and eighty-six of the 968 trees sampled were grafted onto sour orange rootstock. One hundred trees were sampled in 2018; 200 trees were sampled in 2019; 320 trees were sampled in 2020; 4 trees were sampled in 2021; and 344 trees were sampled in 2022. All trees were of fruit-bearing age, from 5 to 25 years of age (data not shown). Grapefruit trees on sour orange rootstock accounted for almost three-fourths of the trees sampled, at 724 trees out of 968 (**Table 3**). We tested sweet orange trees from all three counties, grapefruit from Indian River and St. Lucie counties, and lemon trees from St. Lucie County.

**Table 3:** Summary of citrus tristeza virus (CTV) prevalence in Florida citrus trees during the 5-year (2018-2022) general survey.

County	Scion/Rootstock	CTV strain-positive trees											
		T30	T36	VT/T68	T30,T36	T30,VT/T68	T36,VT/T68		T30,T36,VT/T68		Unidentified strain		Total positive out of total samples
Indian River	Grapefruit (*Citrus paradisi*)¹/Sour orange (*Citrus aurantium*)	2	12	–	–	–	–		–		–		14/424
	Sweet orange (*Citrus sinensis*)²/Sour orange	12	–	–	–	–	–		–		–		12/35
Polk	Sweet orange/Trifoliate orange (*Poncirus trifoliata*)	1	4	4	–	–	6		–		–		15/22
	Sweet orange/Various trifoliate orange hybrids³	5	–	5	–	2	–		–		–		12/32
	Sweet orange/Sour orange	1	–	1	–	–	–		–		–		2/3
St. Lucie	Grapefruit/Sour orange	–	–	–	–	–	–		–		–		0/300
	Sweet orange/Sour orange	2	–	–	–	–	–		–		1		3/100
	Sweet orange/Trifoliate orange	12	2	–	6	–	–		–		–		20/20
	Lemon (*Citrus limon*)^4^/Sour orange	1	–	–	–	–	–		–		–		1/24
	Lemon^5^/US-897	–	–	–	–	–	–		–		–		0/8
**Overall Total Positives**		36	18	10	6	2	6		0		1		**79/968 (8.2%)**

The numbers represent the tree(s) positive for each CTV strain or CTV strain combination detected for each citrus tree type. The dash represents zero detections. ¹Mixture of cultivars: Ray Ruby and Rio Red; ²Mixture of cultivars: Navel, Hamlin, Valencia, and Parson Brown; ³Swingle citrumelo, US-897 (Cleopatra mandarin × Trifoliate orange); ^4^Mixture of cultivars: Harvey and Bearss; ^5^Cultivar Harvey. CTV-positive Ct values generally ranged from 20-30.

Out of a total of 968 citrus trees across three different Florida counties, approximately 8.2% tested positive for CTV with our generic primers. These positive trees were then subjected to strain-specific PCRs, revealing an interesting distribution and assortment of CTV throughout the Florida counties (**Table 3**). There were 14 single infections of either T30 or T36 detected in grapefruit trees on sour orange rootstock out of 424 grapefruit trees tested in Indian River County (**Table 3**). CTV detection in grapefruit was slightly higher compared to 12 CTV-positive sweet orange trees on sour orange rootstock in Indian River County (**Table 3**). Indian River County had comparable detections of T30 and T36 CTV populations. More CTV strains and strain combinations were detected in trees in Polk and St. Lucie counties, specifically in sweet orange trees on trifoliate orange (*Poncirus trifoliata*) rootstocks in both counties (**Table 3**). Similarly, a little over a third of the sweet orange trees on various trifoliate orange hybrid rootstocks tested positive for CTV in Polk County (**Table 3**). Only one of the lemon trees tested positive for CTV in St. Lucie County and none of the 300 grapefruit trees tested positive for CTV in the same county (**Table 3**). One unidentified CTV strain—positive for the generic CTV primers but negative for all strain-specific primers—was detected during the survey and was located in St. Lucie County. Most CTV detections were single infections of the T30 strain, and we never detected all major CTV strains together in one tree (**Table 3**).

In summary, out of the 79 CTV-positive trees detected, 44 trees (approximately 56%) were positive for strain T30, 30 trees (approximately 38%) were positive for T36, and 18 trees (approximately 23%) were positive for VT/T68.

Next, we pooled ACPs to identify the diversity of CTV strains in Florida groves with psyllids. The pooled ACPs sampled during this initial part had more incidences of the T36 strain detected than the citrus tissue sampled and surveyed (**Table 4**). Mixed populations of all the strains screened were detected in numerous samples of the pooled ACPs (**Table 4**). Interestingly, we only detected one instance of an unidentified strain in the ACPs out of a total of 24 pooled samples tested (**Table 4**). The overall total CTV-positive results in ACPs (88%) were much higher than in citrus (8.2%) (**Tables 3**, **4**).

**Table 4:** Summary of citrus tristeza virus (CTV) prevalence and diversity in Asian citrus psyllid (*Diaphorina citri*; ACP) samples tested during the 5-year (2018-2022) general survey.

Florida Region	CTV strain-positive ACP samples								
	T30	T36	VT/T68	T30,T36	T30,VT/T68	T36,VT/T68	T30,T36,VT/T68	Unidentified strain	Total positive out of total samples
A	–	1	–	–	–	2	1	–	4/4
B	1	2	–	1	–	–	–	–	4/6
C	–	1	–	–	–	–	1	–	2/2
D	–	–	–	–	–	–	1	–	1/2
E	–	2	–	–	–	1	1	1	5/5
F	–	2	–	1	–	1	1	–	5/5
**Overall Total Positives**	1	8	0	2	0	4	5	1	**21/24 (88%)**

The numbers represent the pooled ACP sample(s) positive for each CTV strain or CTV strain combination detected. The dash represents zero detections. CTV-positive Ct values generally ranged from 20-30.

### Analysis of citrus tristeza virus in citrus versus Asian citrus psyllid nymphs

In the second part of the study, we compared CTV incidence in citrus trees and nymphs feeding on the citrus leaves. Out of a total of 43 citrus trees—and their feeding nymphs—, CTV was detected in 32 samples (74%) in either citrus tissue or nymphs (**Table 5**). Four citrus trees were positive for CTV, but their feeding ACP nymphs were negative, while three other citrus trees were negative for CTV but had CTV-positive ACPs (**Table 5**). Generally, the same CTV strain detected in the tree was usually detected in the feeding ACP nymphs, and vice versa (**Table 5**), except for two trees. CTV was detected in 29 out of 43 trees (67%) tested using citrus tissue and in 28 pooled ACP samples out of 43 (65%) (**Table 5**).

**Table 5:** Citrus tristeza virus (CTV) and strain detection in citrus tissue or Asian citrus psyllid (*Diaphorina citri*; ACP) nymph tissue during comparative study.

Tree sample ID	CTV detection (strain) from citrus tissue	ACP nymph sample ID	CTV detection (strain) from ACP nymph
T 1	Negative	A 1	Negative
T 2	Negative	A 2	Negative
T 3	Positive (T30)	A 3	Positive (T30)
T 4	Positive (T30)	A 4	Positive (T30)
T 5	Negative	A 5	Positive (T30)*
T 6	Negative	A 6	Negative
T 7	Positive (T30)	A 7	Positive (T30)
T 8	Positive (T30)	A 8	Positive (T30)
T 9	Negative	A 9	Positive (T30)*
T 10	Positive (T30)	A 10	Positive (T30)
T 11	Positive (T30)†	A 11	Negative
T 12	Positive (T30)†	A 12	Negative
T 13	Negative	A 13	Negative
T 14	Negative	A 14	Negative
T 15	Positive (T30)	A 15	Positive (T30)
T 16	Negative	A 16	Negative
T 17	Negative	A 17	Negative
T 18	Positive (VT/T68)	A 18	Positive (VT/T68)
T 19	Negative	A 19	Negative
T 20	Negative	A 20	Negative
T 21	Negative	A 21	Positive (VT/T68)*
T 22	Positive (T36, VT/T68)	A 22	Positive (T36, VT/T68)
T 23	Negative	A 23	Negative
T 24	Positive (VT/T68)	A 24	Positive (VT/T68)
T 25	Positive (T36, VT/T68)	A 25	Positive (T36, VT/T68)
T 26	Positive (T36, VT/T68)	A 26	Positive (T36, VT/T68)
T 27	Positive (T36, VT/T68)	A 27	Positive (T36, VT/T68)
T 28	Positive (T30, T36, VT/T68)	A 28	Positive (T36, VT/T68)
T 29	Positive (T36, VT/T68)	A 29	Positive (T36, VT/T68)
T 30	Positive (T36, VT/T68)	A 30	Positive (T36)
T 31	Positive (T36, VT/T68)	A 31	Positive (T36, VT/T68)
T 32	Positive (T36, VT/T68)	A 32	Positive (T36, VT/T68)
T 33	Positive (T36, VT/T68)	A 33	Positive (T36, VT/T68)
T 34	Negative	A 34	Negative
T 35	Positive (T30)†	A 35	Negative
T 36	Positive (T30)	A 36	Positive (T30)
T 37	Positive (T30)	A 37	Positive (T30)
T 38	Positive (T30)	A 38	Positive (T30)
T 39	Positive (T30)	A 39	Positive (T30)
T 40	Positive (T30)	A 40	Positive (T30)
T 41	Positive (T30)†	A 41	Negative
T 42	Positive (T30)	A 42	Positive (T30)
T 43	Positive (T30)	A 43	Positive (T30)
**Total Positive/Total Tested**	29/43 (67%)	**Total Positive/Total Tested**	28/43 (65%)

T, tree; A, ACP; *Detected in ACP nymphs, but not citrus tree; †Detected in citrus tree, but not ACP nymphs; CTV-positive Ct values generally ranged from 20-25.

Like the sweet orange trees on sour orange rootstock, we detected only T30 in the sweet orange trees on Swingle citrumelo (‘Duncan’ grapefruit × *Poncirus trifoliata*) rootstocks (**Table 6**). The sweet orange trees on a variety of different rootstocks, such as mandarin or mandarin hybrids, consistently tested positive for the strains T36 and VT/T68, and almost always in mixed populations (**Tables 5**, **6**). These mixed populations were simultaneously detected in their feeding ACP nymphs (**Tables 5**, **6**). More CTV strains and CTV in general were detected in the sweet orange trees on a variety of hybrid rootstocks than in sweet orange trees on sour orange rootstock (**Table 6**), like the trend observed in the general survey of trees (**Table 3**).

**Table 6:** Summary of citrus tristeza virus (CTV) strain prevalence in comparative study of citrus trees and Asian citrus psyllid (*Diaphorina citri*; ACP) nymphs.

Tree (Scion/Rootstock)	Tree Sample ID	Citrus positive for CTV (positive trees out of total trees sampled)	ACP Sample ID	ACP nymphs positive for CTV from trees sampled (positive pooled nymphs of 5)	CTV strain(s) detected
Sweet orange ‘Valencia’/Sour orange	T 1 – T 11, T 34 – T 43	15/21	A 1 – A 11, A 34 – A 43	14/21	T30
Sweet orange ‘Valencia’/Swingle citrumelo	T 12 – T 15	2/4	A 12 – A 15	1/4	T30
Duncan grapefruit/Kuharske	T 16 – T 21	1/6	A 16 – A 21	2/6	VT
Sweet orange¹/Various hybrids²	T 22 – T 33	11/12	A 22 – A 33	11/12	T30, T36, VT/T68 (almost always mixed)

T, Tree; A, ACP; ¹Mixture of cultivars: Hamlin and Valencia; ²Wilking mandarin × Trifoliate orange, Cleopatra mandarin × Swingle trifoliate, Sun Chu Sha mandarin, Sunki mandarin × Swingle trifoliate.

## Discussion

Previous surveys for CTV throughout Florida citrus budwood sources and groves provided insight into the distribution of mild and severe strains already present across the state (Cohen and Knorr, 1953; Norman et al., 1961; Garnsey and Jackson Jr., 1975; Garnsey et al., 1980; Brlansky et al., 1986; Yokomi et al., 1992; Garnsey, 1995; Hilf and Garnsey, 2002; Halbert et al., 2004; Sieburth and Nolan, 2005; Harper and Cowell, 2016). These surveys showed that the majority of CTV infections throughout Florida citrus were largely attributed to mild T30 strains or in combination with other strains, with less incidence of severe or decline-inducing strains (Garnsey, 1995; Hilf and Garnsey, 2002; Halbert et al., 2004; Harper and Cowell, 2016). Our more recent CTV survey over five consecutive years (2018-2022) showed the persistent presence of CTV, and similarities, but also revealed some differences compared to prior surveys conducted. The T30 strain was detected in over half the CTV-positive trees and continued to be the dominant strain, but just over one-third of the positive trees had T36 strains in mostly mixed populations. The VT/T68 strain has been historically the least prevalent major strain throughout Florida (Harper et al., 2015b), and continues to be in this current survey, detected in less than a fourth of the CTV-positive trees. A few years ago, Harper and Cowell (2016) also revealed an increasing prevalence of the VT strain across the state, which seems to be a continuous trend according to our survey. Harper and Cowell (2016) found their VT-like isolates limited to Polk and Hillsborough counties, along with single groves in Collier and Marion counties. Similarly, we only detected VT/T68 isolates in Polk County during our initial survey, as well as during our second comparative analysis in Collier County. Florida has been able to evade severe stem-pitting strains in commercial groves; however, our numerous detections of VT/T68 isolates in this study should remind growers about the dynamic and potentially adverse behavior of CTV throughout citrus in this region (Garnsey, 1995; Halbert et al., 2004; Sieburth and Nolan, 2005; Harper et al., 2015b).

In 2014 and 2015, Harper and Cowell (2016) also found a 100% incidence of trees in Polk County infected with mixed T36, T30, and VT populations. We surprisingly did not find any incidence of all three strains in one tree during the initial survey, but we did find one tree infected with all three strains during the comparative investigation (in Collier County). This difference may be of interest to explore in the future. It is possible that trees afflicted with all strains in the past, exacerbated by HLB, were dead and/or removed before our sampling throughout these commercial groves. Increasing the sample size might detect more trees with all three strains too. The incurable HLB epidemic has left >95% of Florida citrus trees infected across the state and may continue to intensify other citrus pathosystems besides CTV, like citrus canker, by disrupting tree physiology (*personal observations*). The perennial production of citrus trees allows for repeated encounters with pathogens year after year, especially in the HLB endemic environment in Florida. The observed tolerance of sour orange to HLB has also brought back interest in replanting trees with this rootstock and its hybrids (Albrecht et al., 2012; Stover et al., 2016; Castle et al., 2020; Bowman et al., 2021). Together this shows the current transforming status of CTV in Florida and stresses the importance of mitigating potentially larger CTV epidemics.

Out of 79 CTV-positive trees during this survey, only twelve trees on sour orange rootstock had T36. This low incidence of the decline-inducing strain on the susceptible rootstock is not too surprising, since all groves surveyed were commercial. Maintenance of the grove and loss of tree profitability would likely result in the removal of a citrus tree on sour orange rootstock if infected with T36 and declining due to CTV and/or HLB. Thus, we did not detect many CTV T36 isolates in trees grafted on sour orange rootstock during our survey.

We discovered more CTV strain combinations in the pooled insects compared to the citrus trees surveyed likely because we sampled ACPs from additional counties (and regions) versus the three counties used for citrus samples. We also tested more ACPs in general, and included mixed samples of adult and nymph ACPs during the first part of the study compared to the second part. The wider collection and combination of adult and nymph ACPs sampled shows a broader picture of the presence of more CTV strains and potential combinations present throughout Florida citrus groves. Mobile adult ACP may fly hundreds of meters and feed on several trees throughout its lifetime (Tiwari et al., 2010), which offers a unique way to sample broader areas and more trees for CTV diversity. Though we did not compare CTV strain diversity between ACP nymphs and ACP adults, CTV strain diversity is likely higher in adults versus nymphs because of the mobility of the adults.

In our comparative CTV study, the simultaneous detection of the same CTV strain in the nymph as in their host citrus tree provides further evidence of ACP uptake of the citrus virus from the infected tree. Discrepancies of four citrus trees positive for CTV but not their feeding ACP nymphs, and three other citrus trees negative for CTV but with CTV-positive ACPs, could have resulted from the variable distribution of CTV in the citrus tissue and the feeding nymphs tested, or detection limitation. CTV is not homogenously distributed throughout the infected plant, so it is possible that we tested citrus with CTV and ACPs from citrus tissue without CTV from the same tree (Lee et al., 1988; Cambra et al., 2002; Bar-Joseph et al., 2010). Likewise, we might have tested nymphs from citrus tissue with CTV, but also tested citrus tissue from the same tree without CTV.

Detecting the same mixed populations of CTV strains in the feeding nymphs collected from their accompanying infected citrus tree suggests that ACPs may non-selectively uptake the CTV strains. All the CTV strains we screened for were detected in CTV-positive ACP nymphs (except two cases) along with their citrus host, which suggests that ACPs can uptake different CTV strains and strain combinations present in the tree. Whether this occurs with individual nymph ACPs on the infected tree is unknown and might be of relevance. For detection of CTV in the ACPs, we used much less ACP tissue (< 1 mg) compared to 100 mg of citrus tissue for each tree, which suggests easier access to phloem contents in the ACP versus citrus petiole tissue—and CTV if present. As a result of these comparable detection rates between substantially different tissue amounts, we explored whether we could extract nucleic acid of equivalent concentration and quality of ACP nymph tissue for PCR analysis using less reagents. We found that by halving reagents for the same amount of ACP nymph tissue, we were able to extract the same and even more nucleic acid of equivalent quality (data not shown) compared to extractions with higher reagent volumes. These results suggest that ACP nymphs could be a faster and more economical way to accurately screen for CTV throughout Florida citrus groves.

Bertolini et al. (2008) found that approximately 19% of single field aphids were positive for the virus and Marroquín et al. (2004) found 19-38% of single field aphids (depending on aphid species) carried CTV. Curiously, CTV was detected in approximately 28% of single field ACPs (Britt, 2021). The citrus virus is hypothesized to be consumed by ACPs as a part of its phloem diet, likely fragmented in the gut of the insect and awaiting further degradation with other dietary contents (Britt et al., 2022). The ACP is similar to the aphid in both size and ecology, ranging between 3-4 mm and laying up to 500-800 eggs per lifetime (Mead, 1977; Hall et al., 2013). Following the length of time CTV and the ACP have been in the state (1952 and 1998, respectively) and the overlap of the pathosystems’ dependence on citrus phloem, viral uptake by the ACP was inevitable (Grant, 1952; Halbert, 1998; Dawson et al., 2013). It is worth emphasizing that—at this time—we cannot definitively say whether CTV is transmitted by the ACP as it is transmitted by the aphid. The variable transmission of CTV by the aphid and a lack of understanding of the citrus virus in the ACP should caution vector competency parallels. There is research attempting to address whether the ACP transmits CTV, yet validation by multiple outlets is lacking, and the concept is still debatable.

During both parts of this project, we detected multiple single strain populations (mostly T30) in sweet orange groves grown on sour orange, trifoliate orange, or Swingle citrumelo rootstocks, which warrants further consideration as to why other strains were not detected. Most CTV infections in field citrus trees throughout the state have been shown to harbor multiple strains (Harper et al., 2015b). We hypothesize that the susceptibility of trees grown on sour orange rootstock to certain CTV strains, such as quick decline from T36 infection, likely led to their loss of profitability (even death) and subsequent removal from the grove before sampling and testing, as mentioned previously. Thus, “mild” T30 infections likely remained in the grove and spread between trees by CTV-positive aphids we had also collected and tested from such groves (data not shown). T30 was the only CTV strain we detected in these aphids. Additionally, these localized populations of T30 in commercial groves may have also resulted from budwood infected with a mild CTV isolate that tested negative for the monoclonal antibody MCA13 (Permar et al., 1990). This assay has been used to screen all registered Florida citrus budwood for severe and decline CTV isolates used in propagation (Hilf and Garnsey, 2002). Though we collected and tested a couple pooled aphid samples during this survey, we did not screen aphids like psyllids because aphid populations were much more sporadic and were not as high or as often encountered as psyllid populations in visited groves. This serves as an additional reason that ACPs (and not the actual vector of CTV) may be a convenient bioindicator of the citrus virus in Florida. It is important to mention that because nymphs are so dependent on citrus flush tissue, the season and health of the citrus tree can influence the efficiency of finding nymphs for CTV testing. Nevertheless, the physiological effects of endemic HLB in Florida have also altered flushing times for many groves, increasing chances of finding flush (and ACP nymphs) many times during the year (Britt et al., 2020, *and personal observations*).

Differences in CTV prevalence between the citrus types, specifically that grapefruit and lemons had much lower CTV versus sweet orange, have also been documented before (Bridges and Youtsey, 1972; Garnsey, 1995). Roistacher and Bar-Joseph (1984) demonstrated poor transmission rates of CTV between lemon and grapefruit hosts, as Roistacher et al. (1984) demonstrated a lower aphid feeding preference of lemon trees compared to sweet orange trees. Alternatively, if infected and unprofitable, these grapefruit and lemon trees might have been removed during grove maintenance before our sampling, as suggested earlier. These persistent and localized T30 populations in commercial groves may represent selective events of mild CTV isolates throughout Florida, yet why severe strains were not present may be of significance. Most of the grapefruit trees sampled during this project (~59%) were from Indian River, because this county has the majority of grapefruit acreage in Florida (NASS-USDA, 2022).

We detected two unidentified strains during the initial survey, one in the ACPs and one in a sweet orange tree on sour orange rootstock, but they were not further investigated. It would be of interest to identify their strain genotypes, along with additional CTV diversity circulating in Florida citrus groves. This knowledge would not only help the industry prepare against the emergence of possible severe isolates, but could also reveal the presence of novel strains previously undetected in this region. Finally, it was not unusual to find more CTV and increased CTV strain diversity in the sweet orange trees on hybrid rootstocks compared to sweet orange trees on sour orange rootstock during this study. The hybrid rootstocks in the first part included crosses with trifoliate orange, and the second part of this research included crosses with mandarin, citrus types known to have more tolerance to CTV infection than sour orange (Bridges and Youtsey, 1972; Krueger and Navarro, 2007; Castle et al., 2020). Thus, longer-living, productive trees on more CTV-tolerant rootstock may accumulate more CTV strains and harbor more CTV diversity in a commercial grove than declining and subsequently removed trees on sour orange rootstock. This may also result from volatiles emitted by certain citrus scion/rootstock hosts and their CTV-infected counterparts, with some more attractive to aphids than others (Pickett et al., 1992; Guarino et al., 2021).

In conclusion, this study demonstrated the continual and diverse presence of CTV throughout citrus groves in Florida by surveying in both CTV-susceptible and -tolerant rootstock/scion citrus varieties, and by exploiting an unforeseen tool: the Asian citrus psyllid. Through this investigation, we utilized the overlap in the ecology of the CTV and HLB pathosystems, which are both reliant on citrus phloem. By discovering the ACP as an unexpected CTV reservoir, we were able to detect similar CTV in the insect and the actual infected host. Using ACPs as a future bioindicator for CTV would be less labor intensive (especially in Florida weather conditions). As growers combat challenges associated with HLB, this and previous recent studies (Harper and Cowell, 2016; Britt et al., 2020; Britt et al., 2022) must remind the citrus industry of the persistence of this citrus virus throughout Florida and the threat it may pose if overlooked during citrus HLB mitigation efforts.

## Data availability statement

Inquiries on data can be directed to the corresponding authors.

## Author contributions

KB-U assisted with psyllid surveys, citrus and psyllid samplings, conducted PCRs, analysed the data, and drafted the manuscript. DT assisted with citrus and psyllid surveys/samplings, conducted PCRs, and analysed the data. PS assisted with citrus and psyllid surveys/samplings, conducted PCRs, and analysed the data. OB and AL received the funds and designed and directed the study. OB and AL identified grove sites, collected citrus and psyllids, analysed the data, and wrote the manuscript. All authors contributed to the article and approved the submitted version.
